# Proximal humerus fractures in the elderly: is there (still) a role for plate osteosynthesis?

**DOI:** 10.1007/s00068-025-02923-6

**Published:** 2025-07-04

**Authors:** Charlotte M. Lameijer, Leanne Blaas, Robert Jan Derksen, Klaus Wendt

**Affiliations:** 1 Department of Trauma Surgery, Amsterdam University Medical Center, Amsterdam, Netherlands; 2 Department of Trauma Surgery, Zaans Medical Center, Zaandam, Netherlands; 3 Department of Trauma Surgery, University Medical Center Groningen, Groningen, Netherlands

**Keywords:** Proximal humerus, Plate osteosynthesis, Eldery

## Abstract

Introduction: Proximal humeral fractures (PHFs) are among the most common fractures in the elderly population resulting from low energy trauma. The primary aim of this review is to assess the role of primary locking plate osteosynthesis in elderly patients with complex PHFs and highlight key surgical techniques to optimize outcomes. A literature overview and two illustrative cases are presented. Conclusion: Treating PHFs in elderly patients requires careful patient selection, adequate patient informed consent and shared decision making. Fit elderly (60–75 years) patients with complex 3- or 4-part PHF may benefit from plate osteosynthesis. For best outcomes, optimal anatomical and stable reconstruction of the medial hinge and tuberosities is mandatory and use of non-absorbable sutures for the rotator cuff is highly important. Augmentation with a fibula strut or cement could be considered to achieve optimal outcomes. In less fit elderly patients, with a complex 3- or 4- part fracture, reverse shoulder arthroplasty (RSA) may be preferable. It is important to provide both plate osteosynthesis and RSA options in practice.

## Introduction

Proximal humeral fractures (PHFs) are among the most common fractures in the elderly population resulting from low energy trauma [1]. Along with the increasing life expectancy of the global population and subsequent osteoporosis, the incidence of these fractures is rising rapidly [2, 3]. PHFs occur mostly in active persons aged 60 years and older [4]. Around 90% of these patients live independently at home and do their own shopping and housework. Hence, a PHF can potentially affect this independence and deteriorate the quality of life of the active elderly patient [5]. For older adults, regaining functional mobility is critical, as it enables them to maintain their quality of life and stay self-sufficient. One of the surgical interventions for managing PHFs is locking plate osteosynthesis, which stabilizes the fracture and promotes bone healing. This approach aims to restore the anatomical structure of the humerus, providing a solid foundation for rehabilitation and allowing the patient to return to their normal routines. Given the aging population and the challenges posed by osteoporosis, understanding the benefits and risks of locking plate osteosynthesis in treating these fractures is essential for optimizing recovery and supporting elderly individuals in living independently. The primary aim of this overview is to determine what the role is of primary reconstruction with locking plate osteosynthesis of a complex PHF in the active elderly patient. In addition, several surgical techniques to optimise outcome following locking plate osteosynthesis are described.

## Indication

**Fig. 1:**
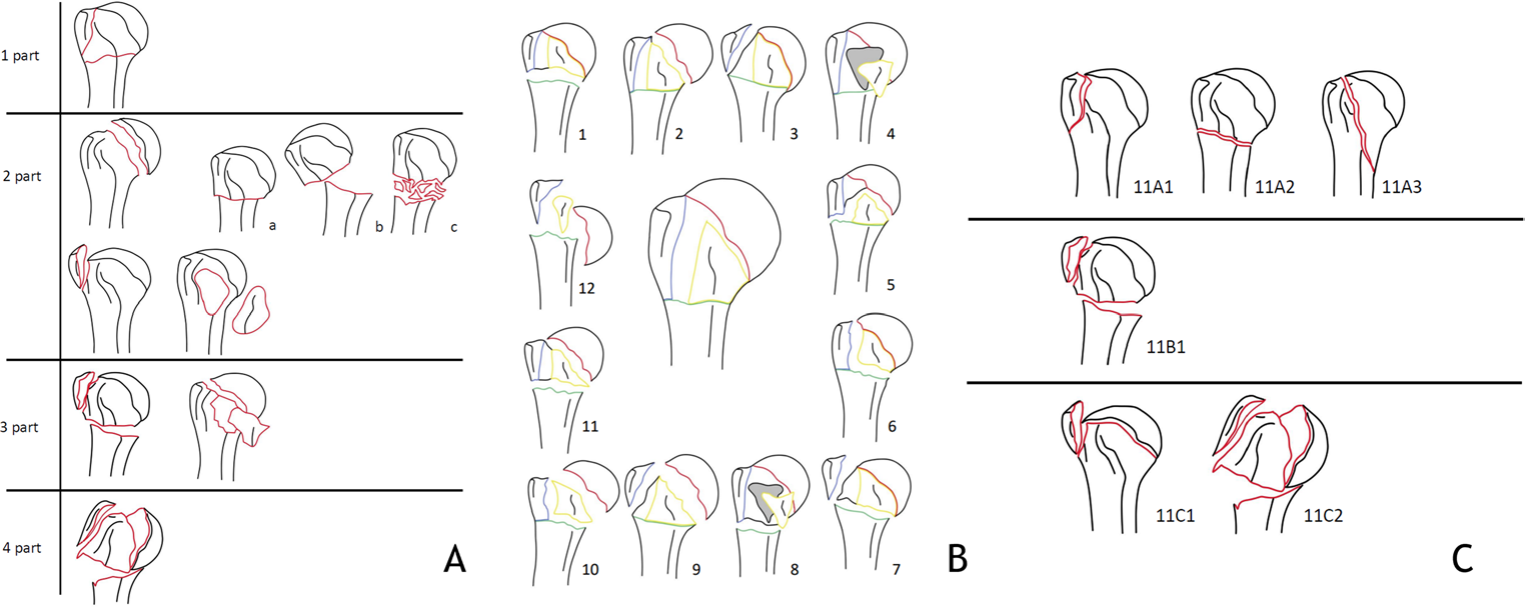
**A**. Neer classification [6]. **B** Hertel classification [7]. **C** AO classification [8]

**Fig. 2:**
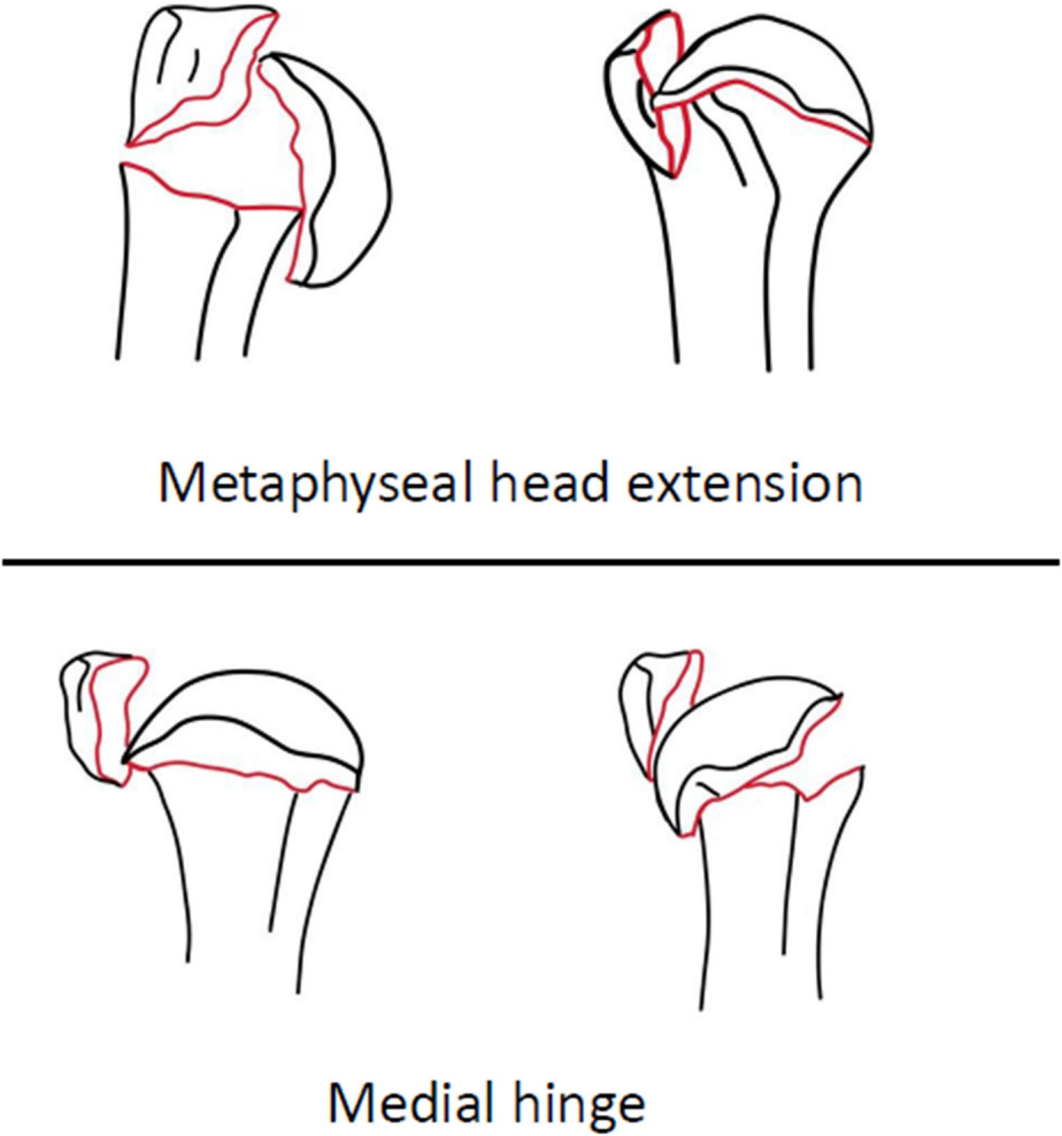
Prognostic factors for avascular head necrosis [7]

PHFs can be classified by the Neer, Hertel or AO- classification as one-, two-, three- or four-part fractures (Fig. 1) [6–8]. To date, research has not been able to identify clear indications for an operative treatment of PHFs. Historically, indication for surgical treatment is based on one or more of the following radiographic factors:1) more than 1 cm dislocation of one or more parts, 2) more than 45 degrees of angulation,3) fractures-dislocations and 4) dislocated head-split fractures [6]. However, prognostic factors for the risk of avascular necrosis (AVN) should be taken into account when indicating surgical treatment; metaphyseal extension < 8 mm, disrupted medial hinge, basic fracture morphology, head split fractures (> 20% head involvement), angular head displacement > 45 degrees, tuberosity displacement > 10 mm, glenohumeral dislocation [7] (Fig. 2). Indication for surgical treatment is becoming more and more individualized based on patient- and fracture characteristics. A practical algorithm to support the decision making for optimal indication, specific (surgical) treatment and outcome of PHFs is needed.

### Treatment

According to Neer in 1970, 85% of proximal humerus fractures are non-displaced and treated non-surgically [9]. However, more recent studies suggest significantly higher prevalence of displaced PHFs [10, 11]. In addition, many studies suggest conservative treatment for displaced, complex PHFs is preferred for older, low-demanding patients or patients with many comorbidities [9, 12, 13]. They argue that conservative treatment is more cost-effective and less invasive with potential good functional outcomes. In case of dislocated proximal humerus fractures, a wide range of surgical treatment options is available. The frequently used surgical options consist of (1) open reduction and internal fixation with locking plate osteosynthesis, (2) intra-medullary nail fixation (3) reverse (fracture) shoulder arthroplasty (RSA) and (4) hemiarthroplasty [12].

Locking plate osteosynthesis provide divergent and convergent fixed-angle screws that improve fixation and pullout strength [14–16]. However, complication rates as high a 36% have been described [14, 17–21]. The most common complications include secondary loss of reduction indicated by either screw cutout, penetration of screws though the humeral head and/or varus collaps of the humeral head. These are associated with compromised vascularization of the humeral head which results in union problems and avascular necrosis (AVN) of the humeral head [7, 22–24]. These complications occur predominantly in complex fracture types, often including an anatomical neck fracture of the humerus [21, 25, 26]. Because a fracture of the anatomical neck of the proximal humerus is oblique, vertical shearing forces may promote secondary displacement when lateral plate fixation is used without reinforcement [14, 15, 22, 23, 25]. A solution to prevent secondary displacement and possible AVN lies in establishing sufficient medial hinge support with an allograft in addition to the locking plate construct [15, 22]. Another option is cement augmentation of the plate osteosynthesis [27, 28].

A shift towards primary placement of Reversed Shoulder Arthroplasty (RSA) for complex, displaced PHFs in the elderly, is taking place and promising outcomes have been reported [29]. The advantages comprise of the offset of the ball and socket by using the RSA; through medialization of the center of rotation, the shoulder function improves by extending the lever arm for the deltoid muscle. With this mechanism, the functionality of the arm is less dependent on the rotator cuff [30]. However, it should be taken into consideration that the shoulder function is grossly dependent on the deltoid muscle with RSA. Optimalisation of the deltoid muscle by inverting head and socket (Grammonts’ principle) does however diminish the optimal function of the rotatorcuff, since the center of rotation is not central to the cuff insertions anymore. In addition, fatigue of the deltoid can be a problem in the long term. RSA is generally seen as the last resort and is therefore often used with precaution or as a salvage procedure. Nonetheless, RSA as primary treatment for displaced PHFs is upcoming as favorable over secondary RSA after failed treatment. The tubercles can be reinserted in primary treatment, leading to better functional outcomes. In secondary surgery, the tubercula are often unavailable or the rotator-cuff is atrophied, leading to worse functional results. Furthermore, surgery after failed conservative or surgical treatment is more demanding due to the inexistence of normal anatomical landmarks, the need to revive the edges of the fracture, and the removal of previously placed hardware [29]. Placement of RSA is challenging, either in primary and especially in secondary placement following PHFs, and a learning curve of approximately 20 procedures has been reported to achieve steady outcomes [31]. Also, as mentioned earlier; fatigue of the deltoid and diminished rotatory function of the cuff muscles with risk of scapular notching can diminish function in the long run. No reliable studies are present regarding learning curve for the reconstruction of PHFs. For complex PHFs, this might even be more challenging and learning curves might exceed 20 procedures.

The question arises which active elderly patients with dislocated complex 3- and 4-part PHFs should be treated with a plate osteosynthesis or a primary reconstruction and how? Should (biological) age, negative fracture characteristics, patient informed consent and shared decision making be a part of this. And should the shoulder surgeon not be able to convert during surgery when primary reconstruction is not feasible? This review aims to give an overview and advice regarding these questions.

## Surgical technique

### Approach

**Fig. 3:**
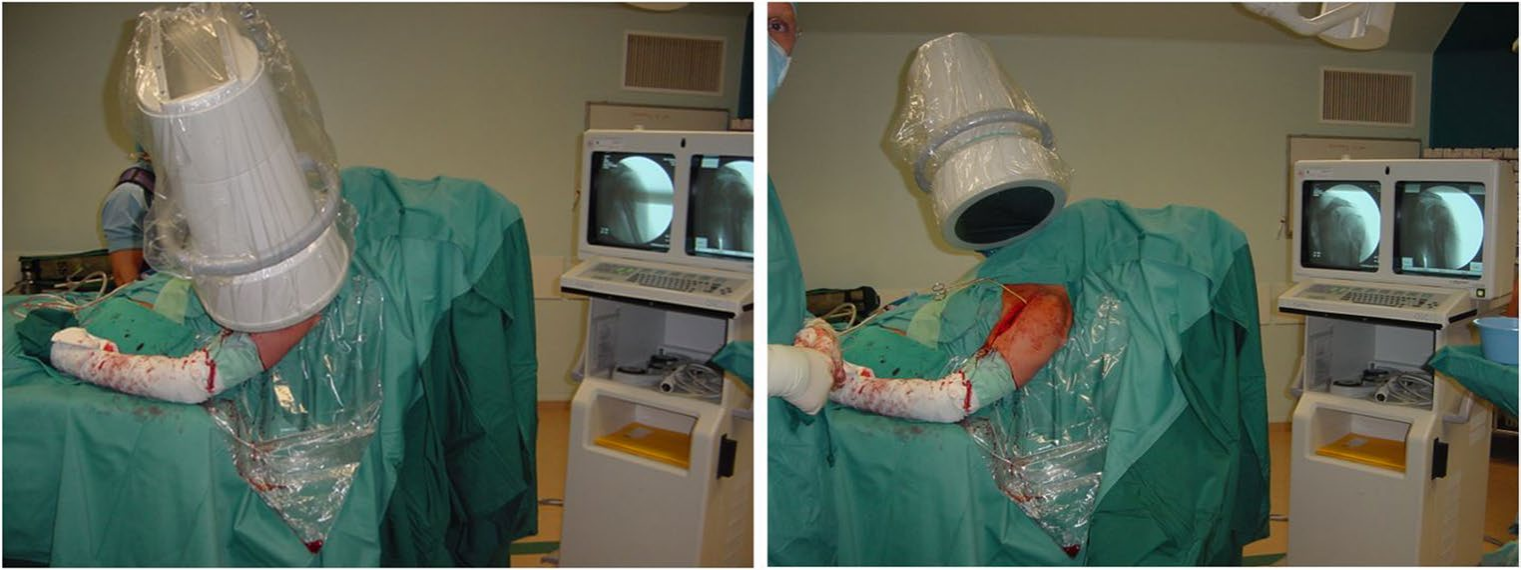
Beach chair position and optimal positioning of the image intensifier

The procedure is performed in a supine or beach chair position on a radiolucent table (Fig. 3). An image intensifier is necessary. There are two surgical approaches: the deltopectoral approach and the deltoid split. The latter has been described to be less invasive. However, the deltoid muscle is very important in the rehabilitation following PHFs, because the cuff can be (temporarily) insufficient. The axillary nerve, which is responsible for the innervation of the deltoid, is at risk during the deltoid split approach and care must be taken not to compromise it. There are no differences in clinical and radiological outcome between the two approaches, even in complex 3- and 4 part proximal humerus fractures, but the operation time is reported to be significantly shorter in the less invasive deltoid split approach [32–35]. However, selection bias may be present, due to the fact that the more complex PHFs might mostly be approached through the deltopectoral approach influencing operation time negatively. Expertise of the shoulder surgeon is leading in choosing the surgical approach. However, when operating complex 3- and 4-part PHFs with plate osteosynthesis with high risk of primary or secondary conversion to RSA, it might be considered to use the deltopectoral approach to protect the axillary nerve.

Meticulous anatomical and stable reconstruction should be strived for to diminish the risk of complications such as secondary dislocation and AVN. Perioperative tricks to reconstruct anatomical are placement of temporary K-wire fixation of the humeral head in the glenoid while reconstructing anatomically. In addition, refixation of the separate cuff muscles and attached tubercula to the plate with 3 nonabsorbable sutures add to the mechanical stability and unloading of forces on the locking screws with little purchase in the humeral head. In addition, cannulated screws or headless compression screws (or small additional plates) can be used through a separate window to obtain optimal anatomical reconstruction and stability [36].

### Implant choice

For 2-part PHFs with surgical neck, outcome for patients aged 65.41 years (± 7.49) treated with a plate osteosynthesis in comparison with intramedullary nailing, did not differ statistically significantly with regards to Constant and VAS scores or radiological final NSA [37]. However, longer operation duration (102.8± 25.65 min versus 86.67 ± 23.39, *p* =.012) and perioperative blood loss (250 versus 175 ml, *p* <.001) was reported as longer union time for the plate fixation group (12 versus 10 weeks, *p* =.003). The question arises if these statistically significant differences are clinically relevant.

Although some authors describe reconstruction with intramedullary nail for dislocated 3- and 4-part PHFs, in general the implant of choice for primary reconstruction is an angular stable locking plate [38].

### Screw characteristics

Several studies have underlined the importance of screw trajectory and number of screws to reduce the screw cut out risk in plate osteosynthesis for complex 3- and 4- part PHFs [39–42].

In clinical setting, in a cohort of 121 patients (age 64.4 +/- 15.4 years), the use of one medial support screw less than 5 mm of the subchondral bone in the inferomedial quadrant of the humeral head, showed no inferior outcomes than 2 medial support screws. with regard to functional outcome (Constant-Murley score), time to union, loss of neck-shaft-angle (NSA) or humeral head height (HHH). Also the incidence of complications did not differ between one or two medial support screws. However, insufficient reduction following surgery (NSA < 125°) was found to be a significant risk for surgical complications. Other studies have underlined the importance of optimal screw trajectories to prevent screw cutout. Especially virtual studies, using finite element techniques have shown that optimal (patient- and fracture specific) placement of screw trajectories will diminish the risk of screw cutout [40, 42] Therefore, it might diminish complications such as secondary dislocation and subsequent AVN. In these finite element models, also purchase of screws in osteoporotic bone have shown to be more optimal with optimalisation of the screw trajectory. Although this seems a promising method, in order to be able to use this in the clinical setting, more research is mandatory on patient/fracture specific planning of screw trajectories with 3D imaging.

### Fibula strut augmentation

Good outcomes have been reported using fibula allograft augmentation in complex displaced PHFs in active patients [16, 17, 20, 25, 43–48]. The allograft acts as a medial strut across the fracture site and supports the articular head preventing varus malalignment and creating a more stable construct by supporting the medial hinge without compromising the viability of the articular surface [15–18, 49]. In addition, the working length of the screws diminish, due to additional purchase in the fibular strut. This technique appears to reduce the number of complications and enhances the functional outcome in dislocated anatomical neck PHFs [14, 15, 17, 18, 20, 45, 50–52]. Generally, two ways of implementing the fibula strut for PHFs is described; it can be used as an intramedullary bone peg or as a transverse strut to support the medial hinge (Fig. 1). One recent systematic review describes 10 included studies with 802 patients (mean age ranged 59.9–73.3) in which 366 patients were treated with an LCP augmented with fibula graft and 436 were treated with an LCP fixation. Patients treated with augmentation of a fibula graft had significantly better functional outcome with regards to the American Shoulder and Elbow Surgeons (ASES) score (WMD = 5.08; 95% CI 3.69–6.48, *p* <.00001), although the Constant-Murley scores were not significantly different between both groups. Odds ratio for developing a major complication was better in the augmented population (OR = 0.37; 95% CI, 0.23–0.59, *p* <.0001). In addition, radiological measurements, such as change in humeral head height and neck-shaft-angle did not change as much in the augmented group as in the non-augmented group (respectively WMD=−2.4, 95%CI, −2.49- −2.31, *p* <.0001 and WMD=−5.71, 95% CI, −6.69 - −4.72, *p* <.0001) [48].

### Cement augmentation

As discussed earlier, anatomical reconstruction and stable fixation is key to prevent complications. The challenge is to guarantee a flexible fixation to unload the bone-screw interface, but also a rigid fixation to prevent fracture movement [53]. Several studies report on augmenting the screws with cement [27, 28]. A systematic review, including 8 studies studies involving 635 (334 augmented) patients, report reduced implant failure rate (odds ratio (OR) = 0.19; 95% confidence interval (CI) 0.10–0.39; *P* <.0001) and total complication rate (OR = 0.45; 95% CI 0.29–0.69; *P* =.0002) and improved DASH scores (mean difference (MD) = 2.99; 95% CI 1.00–4.98; *P* =.003) in comparison to no cement augmentation [28]. However, there was no significant difference in clinical outcomes, including revision rate, avascular necrosis rate, and constant score. Another systematic review published around the same period, report similar results of 6 included studies with a total of 541 patients (mean age ranged 58–76 years) [27]. The overall complication rate was significantly lower in the augmented group (15.6% versus 25.4%, OR 0.54 (95%CI 0.33–0.87)). This was caused by a reduction of implant-related complications (10.4% vs. 19.9%, OR 0.49 (95%CI 0.28, 0.88)). No difference in AVN was found, although theoretically cement augmentation might cause subchondral necrosis. Data on re-intervention, hospital stay, and operation time was limited but did not show significant differences. No impact on functional scores and general quality of life was detected.

### Patient characteristics

The question remains which elderly patient is fit enough to be indicated for a primary locking plate osteosynthesis and which elderly patient is better treated with a primary RSA, when having sustained a dislocated 3- or 4- part PHF. Although literature is growing more robust on this subject, a solid answer cannot be given [13, 29, 54, 55]. The only study included in the Cochrane overview discussing RSA and primary plate osteosynthesis in elderly patients with 3- and 4-part PHFs, is the DelPhi study [54]. The authors describe a multicenter single-blinded randomized controlled trial including 39 patients receiving RSA and 26 patients with primary plate osteosynthesis of which 90% is female. The Oxford Shoulder was statistically better for the RSA group (respectively 43 versus 38, mean difference 5, *p* =.003). However, the mean difference did not exceed the minimal important change (MIC) reported in literature [56, 57]. Younger patients (ages 65–74) and more complex 4-part fractures had statistically more benefit from an RSA with statistically significantly better Constant Shoulder Score which also exceeded the MIC indicating a clinically relevant difference [57]. However, these optimistic results should be interpreted with caution, because the Constant Shoulder Score is an outcome score which includes a combination of patient reported outcome and range of motion and strength, which are measured by clinicians. These clinician reported outcomes are known to have reasonable inter-observer variability [58]. In addition, the Constant Shoulder Score is known to overestimate outcomes in females > 40 years of age and lower reference values are reported to be used for different age groups, which was not performed in the described study [59]. The same research group published on cost-effectiveness and quality of life regarding the same population, which showed no significant differences in quality of life and costs following RSA or primary locking plate osteosynthesis [55].

Another research group reporting on outcome following surgical treatment of PHFs in elderly patients often referred to, is the PROFHER trial [12, 60]. In a pragmatic randomized controlled trial, including 33 centers, patients were included when ‘the dislocation was *‘sufficient for the treating surgeon to consider surgical intervention*,* but did not have to meet the displacement criteria of Neer’* [12]. The study did not distinguish complex PHFs from mildly displaced fractures. Also, many PHFs were excluded for conservative management without clear criteria. Patients were randomized 1:1 for surgical treatment (106 patients, mean 66.2 years) versus conservative treatment (109 patients, mean 65.8 years). Of the surgically treated patients, 90 patients (82.6%) received a plate osteosynthesis, while 4 patients had intramedullary nailing, 10 received hemi arthroplasty and 5 were treated otherwise. Interestingly, in the participating centers, there were 66 treating surgeons. This resulted in 1.4 locking plate osteosyntheses per treating surgeon (and 2.7 locking plate osteosyntheses per center) in the study period. One might conclude that inclusion was difficult to achieve and selection bias might be present. Another conclusion drawn might be that the expertise per surgeon might not be optimal with these numbers, as surgical treatment for PHFs is challenging. The PROFHER study reported no significant or clinically relevant differences in the outcome as reported with the Oxford Shoulder Score at 2 and 5 years follow-up between the surgical and conservatively treated patients (mean differences of respectively 0.8 and − 1.1) [12, 60]. In addition, complications, secondary surgery did not differ statistically significantly between surgical and conservative treatment. The PROFHER-trial has risk of selection bias due to the selection criteria, homogeneity in surgical treatment, heterogeneity in treating centers and surgeons, and therefore we believe the results of this study should be interpreted with caution.

### Patient reported outcome

Different Patient Reported Outcome Measures (PROMs) have been developed for upper extremity injuries and used for patients with PHFs, with variable content and measurement properties [57]. Frequently used PROMs for patients with PHFs are the Disability of the Arm, Shoulder and Hand (DASH) questionnaire, the Quick-Disability of the Arm, Shoulder and Hand (Q-DASH) questionnaire [61], the Oxford Shoulder Score and the Constant Shoulder Score [62].

The DASH and quick-DASH, as the Oxford Shoulder Score, are PROMs which are constructed according to stringent rules to ensure adequate construct and validity. Following this process, these PROMs have been extensively validated in patients with PHFs [57, 62–64]. However, the Constant Shoulder Score is a combined score of clinician reported outcomes (i.e. range of motion and grip strength), which are known to have moderate intraobserver variability [57]. Also, it was designed in the 1980 s, when knowledge regarding optimally constructing a PROM instrument were minimal. In addition, the Constant Shoulder Score is known to overestimate outcomes in females > 40 years of age and lower reference values are reported to be used for different age groups [59]. When reporting on outcome following PHFs, the (q)DASH or the Oxford Shoulder Score are suitable PROMs, whereas the Constant Shoulder Score is not advisable.

Still, there is variation in the psychometric properties of PROMs, and the concepts measured are not always well defined [65–68]. In addition, completing these traditional PROMs is time consuming for patients and interpretability of the scores and changes in scores is sometimes unclear. To overcome these shortcomings, the Patient-Reported Outcomes Measurement Information System (PROMIS) initiative developed the PROMIS Upper Extremity v2.0 item bank (PROMIS-UE v2.0). This item bank includes 46 questions (items)that all measure one construct and that are ordered on an underlying metric using item response theory (IRT), and designed to be used as short form or Computerized Adaptive Test (CAT) [69]. After answering one item, a computerized algorithm selects the next item from the item bank to be answered. With a maximum of 4–7 items, a reliable and comparable score is produced [70]. The PROMIS-UE v2.0 CAT might be a promising tool for measuring upper extremity function following PHFs. However, further research into patients with PHFs and the PROMIS UE v2.0 is mandatory. When reporting on outcome following PHFs, the (q)DASH or the Oxford Shoulder Score are suitable PROMs.

### Case descriptions

#### Case 1– Active elderly patient treated with locking plate osteosynthesis and fibula strut augmentation

**Fig. 4:**
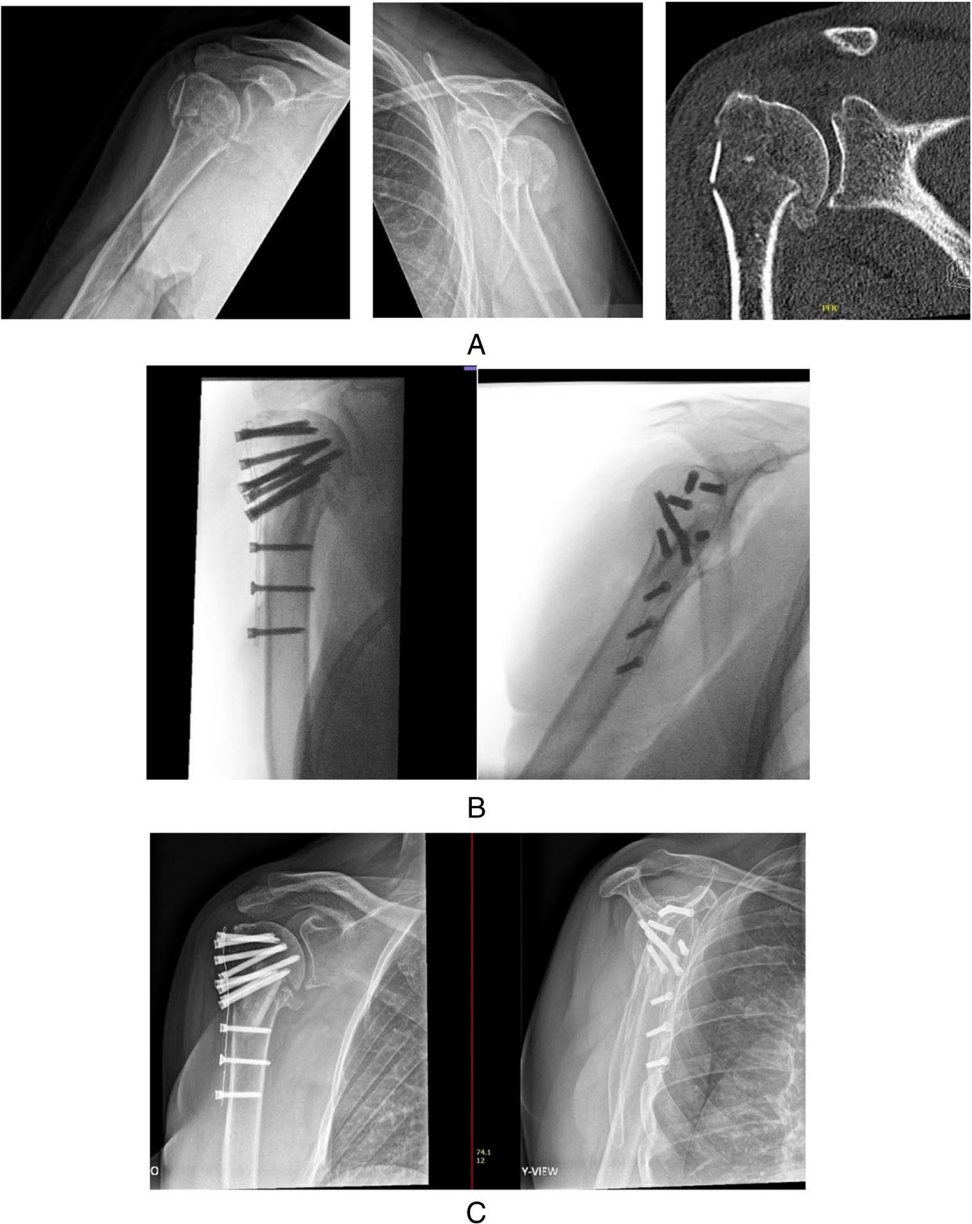
A 62 year old female with a 3-part PHF. **A** Fracture radiographic imaging and CT imaging, **B** Fluoroscopy image during surgical reconstruction, **C** Progressive consolidation at 9 months follow-up

A 62 year old woman presented with a 3-part PHF following a fall from her citybike (Fig. 4A). She had no previous medical history, did light sedentary work and had an active sporting life with swimming and cycling. Following patient shared decision making, a primary reconstruction with locking plate osteosynthesis was chosen. Through a deltopectoral approach, an anatomical reconstruction was performed with locking plate osteosynthesis (Carbofix©) and augmentationwith a fibular allograft. In addition, refixation of the cuff with three non-absorbable onto the plate was performed (Fig. 4B). Postoperatively, she underwent extensive shoulder specific rehabilitation and at 9 months, she had an active range of motion (ROM) of flexion 120°, abduction 110°, external rotation 40° with a PROMIS UE v2.0 CAT score of 47. No complications or signs of AVN were present.

### Case 2- Elderly patient with failed locking plate osteosynthesis treated with secondary placement of an RSA

**Fig. 5:**
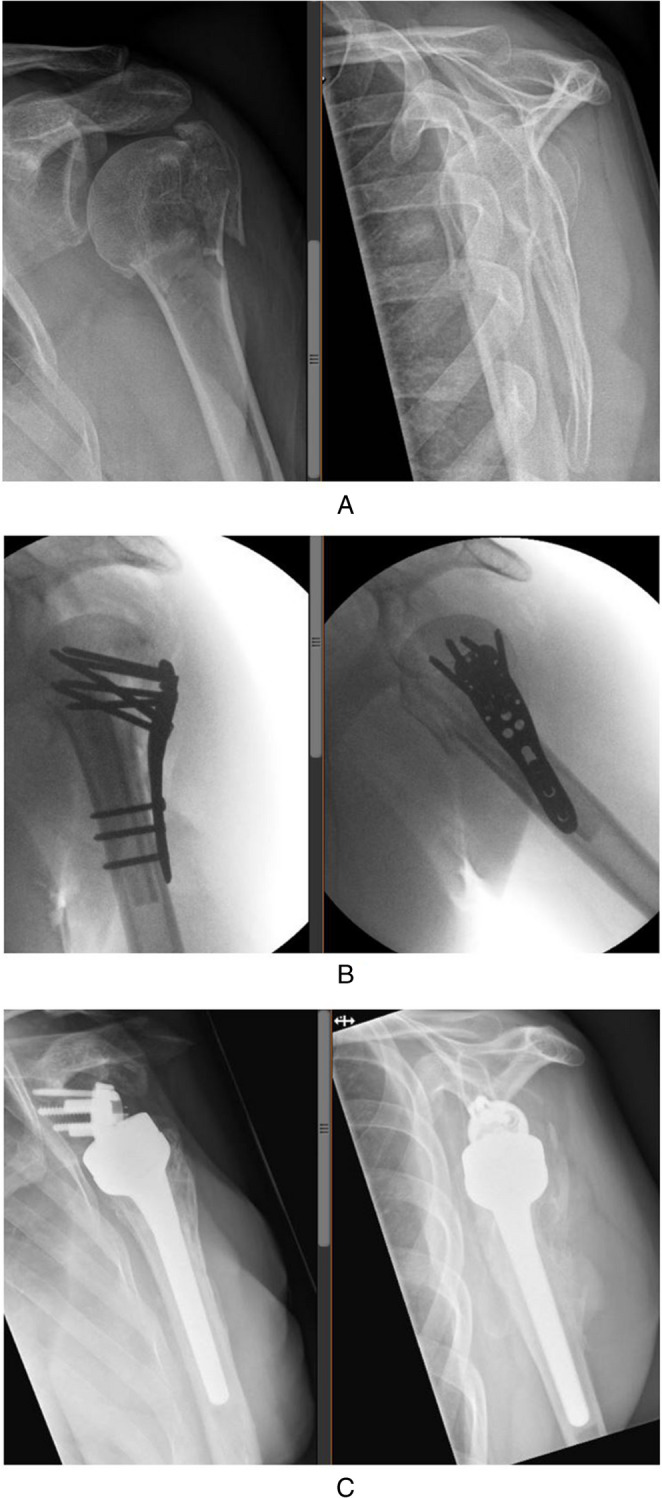
A 71 year old man with a 3-part valgus impacted PHF. **A** Fracture radiographic imaging. **B** Fluoroscopic images of osteosynthesis. **C** Radiographic imaging at final follow-up (18 months after RSA)

A 71 year old man sustained a 3-part PHF (valgus impacted with superiorly dislocated greater tuberosity) following a trip-and-fall on the left shoulder (Fig. 5A). His medical history contained a partial nephrectomy for renal cell carcinoma, a cystoprostatectomy for urothelial cell carcinoma of the bladder and non-insulin dependent diabetes. No neurological deficit following injury was seen. Surgical treatment was indicated and a primary reconstruction with locking plate osteosynthesis was the procedure performed. Via a deltopectoral approach, reduction was conducted with augmentation using a fibular allograft (the graft was inserted with the intent to hold up the valgus impacted articular part of the head (anatomical neck fracture))(Fig. 5B). Postoperatively, the patient underwent an intensive rehabilitation program guided by a shoulder physiotherapist. At 9 months postoperatively, he had regained only 50 degrees of active flexion, 60 degrees of abduction, external rotation of 10° and internal rotation of 10 degrees. Also, the patient still experienced pain when actively using the arm (VAS 5). The patient was referred to a center specialized in PHF sequelae where on ultrasound a partial full thickness rupture of the subscapularis tendon, a total full thickness rupture of the infraspinatus and an absent supraspinatus tendon were seen. Also, the articular head was found to be malunited in slightly too much valgus despite the fibula strut graft. This combination of findings was thought to explain the residual lack of active range of motion and the pain. An indication for plate and strut removal followed by RSA implantation and infraspinatus reinsertion was set and performed (Fig. 5C). No post-operative complications were reported and again a 6-month period of intensive shoulder rehabilitation ensued (first 6 weeks active external rotation was limited because of the infraspinatus repair). On final follow-up (18 months after RSA placement), the patient displayed a nearly full active ROM (abduction 160°, flexion 170° and external rotation 40°). Also, the patient reported these motions to be pain free.

## Conclusion

Understanding of the physical condition of patients and adequate patient informed consent and shared decision making is key when treating elderly patients with PHFs. The active elderly patient between 60 and 75 years of age with a complex 3- or 4-part PHF may benefit from plate osteosynthesis, providing there is an adequate rotator cuff and there are no signs of pre-existent omarthritis. Optimal anatomical and stable reconstruction of the medial hinge and tuberosities is mandatory. In addition, refixation of the cuff with non-absorbable sutures to the plate is highly important. Finally, augmentation with either a fibula strut or cement is known to optimize outcome and should be considered. However, if perioperatively the head seems less viable or optimal anatomical reconstruction cannot be achieved, conversion to an RSA should be possible. The less active elderly patient may benefit from primary surgical treatment with an RSA for a dislocated 3- or 4-part PHF. It is mandatory to provide both surgical options or have a system in his/her practice to provide both plate osteosynthesis and RSA.

## Data Availability

No datasets were generated or analysed during the current study.
